# Development of Organ-on-a-Chip System with Continuous Flow in Simulated Microgravity

**DOI:** 10.3390/mi15030370

**Published:** 2024-03-09

**Authors:** Arnis Strods, Karīna Narbute, Valērija Movčana, Kévin Gillois, Roberts Rimša, Patrik Hollos, Fēlikss Rūmnieks, Arnita Spule, Gatis Mozoļevskis, Arturs Abols

**Affiliations:** 1 Latvian Biomedical Research and Study Centre, LV-1067 Riga, Latvia; karina.narbute@biomed.lu.lv (K.N.); valerija.movcana@biomed.lu.lv (V.M.); felikss.rumnieks@biomed.lu.lv (F.R.); arturs@biomed.lu.lv (A.A.); 2 CellboxLabs Ltd., LV-2164 Ādaži, Latviaroberts.rimsa@cellboxlabs.com (R.R.); arnita.spule@cellboxlabs.com (A.S.); gatis.mozolevskis@cellboxlabs.com (G.M.); 3 Litegrav Ltd. (Litegrav. AI OU), 10134 Tallinn, Estonia; patrik.hollos@litegrav.ai; 4 Faculty of Materials Science and Applied Chemistry, Institute of General Chemical Engineering, Riga Technical University, LV-1048 Riga, Latvia

**Keywords:** microgravity, organ-on-a-chip, continuous flow

## Abstract

Organ-on-a-chip (OOC) is an innovative microfluidic device mimicking the structure and functionality of real tissue. OOCs typically involve cell culture with microfluidics to emulate the biological forces of different organ tissues and disease states, providing a next-generation experimental platform. When combined with simulated microgravity conditions, such as those produced by random positioning machines, they offer unique insights into disease processes. Microgravity has been shown to affect cellular behaviors, like proliferation and viability, though its influence on cell physiology is not fully explored. The primary objective of this study was to develop an OOC model with continuous flow under simulated microgravity. Cells cultured in static (non-continuous-flow) conditions exhibited clear growth reduction under microgravity conditions, showing more pronounced difference compared to continuous-flow conditions using an OOC setup. Although our results show that A549 cell viability under continuous flow decreased in microgravity compared to normogravity, this study demonstrates the successful development of a system capable of providing continuous flow in organ-on-a-chip (OOC) models within a random positioning machine.

## 1. Introduction

It has been shown that the body undergoes significant changes under microgravity conditions, for example, gravity modulates phenotypic transitions at the cellular level occurring both in physiological settings and pathological settings [1]. Although microgravity has notable impacts on cell behavior, including proliferation and viability, its influence on cells remains not thoroughly understood [2]. Additionally, experiments conducted in microgravity have led to the discovery of potential drug targets for various diseases. For example, a new drug that promotes bone formation, developed based on experiments in microgravity, has shown promising results in addressing bone loss due to spaceflight conditions, with implications for osteoporosis treatment on Earth [3]. Therefore, disease research and drug testing in microgravity could discover new targets for drugs or drug resistance development pathways. However, experiments in space are constrained due to limited opportunities and resources. As a result, random positioning machines (RPMs) were designed to simulate microgravity or near-zero-gravity conditions. RPM makes an object rotate around two separate axes, creating a “vector-averaged gravity” environment. RPMs recapitulate genuine microgravity, as these tools continuously alter the gravity vector and the object’s relative position, especially when rotating faster than the speed the object perceives gravity. To prevent centrifugal forces, the RPM turns at a minimal angular speed, ensuring objects at its center remain stationary [4]. Although RPMs cannot fully replicate space conditions, their simulated microgravity is beneficial for studies in fields like cancer research, stem cell treatments, tissue development, drug target identification, and regenerative medicine [5,6], and is the preferred testing method at the European Space Agency (ESA) [7]. In recent studies, exposure to microgravity induced rapid migration and proliferation of lung cancer cells (A549). Under microgravity conditions, these cells also changed their cellular morphology to granular, 3D-like, morphology [8,9]. These findings suggest that microgravity can have complex and different effects on different cancer cell behaviors; therefore, this offers a novel perspective in cancer research.

This research accentuates the importance of dynamic 3D in vitro research models in gravitational forces to understand new aspects in tumor biology.

Organ-on-a-chip (OOC) is a microfluidic device consisting of channels continuously flown with fluids, wherein living cells simulate the structure, interactions, and environment of real tissue and organ functions [10]. This differs significantly from traditional 2D or 3D culture systems due to the shear stress exerted onto the cells thanks to the Poiseuille flow profile in the microfluidic channels. One of the most common setups is based on two vertically stacked channels, separated by a semipermeable membrane. Prior to cell culture seeding, the membrane is covered with an appropriate extracellular matrix (ECM) to ensure a more physiologically relevant environment. The possibility to use different cells in OOC technology makes it particularly useful for modeling specific diseases. Given the discrepancies observed across species in preclinical research, OOC gains added importance. Furthermore, it offers an avenue for drug testing alongside conventional in vitro tests and animal research [8]. OOC tools can enhance precision in early drug screening and better predict a drug’s effectiveness, potential risks, and behavior in humans. Utilizing OOC models in space allows for the rapid study of processes that might take years on Earth [11].

By employing a variety of methods from different disciplines, we might be able to discover new aspects of cancer biology; however, we still need to improve engineering solutions for continuous-flow OOC in microgravitational forces. Therefore, the aim of this study was to develop and demonstrate a proof of principle of OOC with continuous-flow application in microgravity conditions generated by RPM.

## 2. Materials and Methods

### 2.1. Simulated Microgravity Machine

**Figure 1:**
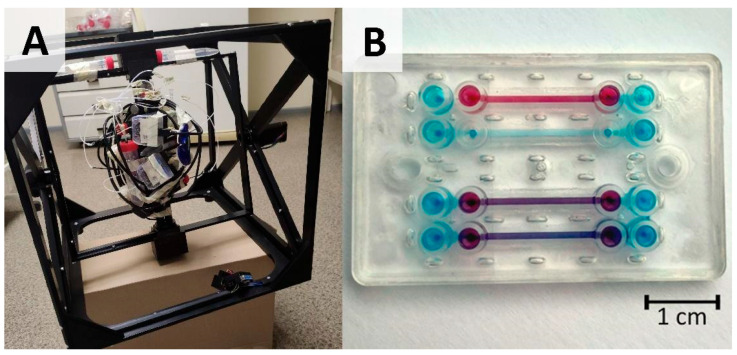
OOC setup for cell cultivation under microgravity conditions: (**A**) image of microgravity simulator (dimensions of frame are 40 cm × 40 cm × 40 cm) combined with pressure-driven OOC cultivation setup adapted for continuous rotation; (**B**) image of microfluidic chip (dimensions are 4.9 cm × 3 cm and total height 6 mm) with bottom channel filled with blue dye and top channel filled with red dye.

A 3D MSRC (microgravity simulator research cube) supplied by Litegrav (Tallin, Estonia) was used in 3D clinostat mode with static path distribution and axial speeds optimized to provide homogenous pseudo 0 g distribution to the sample with the least disturbance to cellular processes, e.g., to shear stress [12]. This mode provides approximately 0.003–0.004 g “zero-averaged” simulated microgravity. The working principle of a simulated microgravity machine is depicted in the Graphical Abstract with an image of the actual setup shown in Figure 1A.

To measure ”sample-perceived” time-averaged g, an accelerometer was mounted close to the center point of the sample plate holder. Acceleration values were recorded for every 3 s over a period of 6 h trials. Time-averaged g forces were calculated by the sum of the XYZ vector averages within time windows, divided by the normalized 1 g control baseline, acquired from stationary device readouts.

As a part of the simulated microgravity setup, a Cellbox Labs proprietary pumping solution was used to ensure continuous flow under microgravity conditions. The flow solution consisted of a high-precision pressure-driven vessel that delivers a user-set over-pressure with respect to ambient pressure, thus driving the culture medium flow in the channel system, ensuring a multi-day culturing setup.

### 2.2. Chip Device Development

Vertically stacked design microfluidic chips shown in Figure 1B were provided by Cellbox Labs (Ādaži, Latvia). The chip design includes a 1.25 mm high top channel, a 0.2 mm high bottom channel, with 1.0 and 1.1 mm channel widths accommodating alignment errors. The overlapping area between the top and bottom channels is 18 mm^2^. Chips are made from cyclic olefin copolymer (COC) formed via injection molding and a track-etched polyester (PET) membrane with 3 µm pore size, and pore density of 0.8 × 10^6^ pores per cm^2^. Connections to the chips were ensured via standardized mini-Luer connections with a 384-well pitch between the inlets.

### 2.3. Cell Culturing

Cells were kept in a controlled environment at 37 °C in a humidified atmosphere containing 5% CO_2_. Cell medium was changed every 2 days.

A549 (human lung adenocarcinoma alveolar basal epithelial cells; CCL-185) were obtained from ATCC and cultured in high-glucose DMEM medium (41,965,062, Gibco, Taufkirchen, Germany), supplemented with 10% iFBS and 0.1% primocin. Passages 15–25 were used for experiments.

### 2.4. Cell Cultivation Using 24-Well Plate

A549 cells were seeded on a 24-well plate (Corning, New York, NY, USA) in triplicate in three various amounts—2000, 4000, and 10,000 cells per well, leading to a density of 1075, 2150, and 5376 cells/cm^2^, respectively—followed by overnight incubation at 37 °C in a humidified atmosphere containing 5% CO_2_ to allow cell attachment. Before connecting the plate to the inner frame of the microgravity device, the wells were filled with cell medium up to the brim and thoroughly sealed with Parafilm^®^ M (Bemis, Neenah, WI, USA).

An identical plate was prepared and incubated in a CO_2_ incubator at 37 °C to serve as a normogravity control.

### 2.5. Cell Seeding into Chip Devices

Prior to cell seeding, chip devices were sterilized under a UV lamp within a laminar flow hood, followed by an additional 30 min sterilization using a 70% ethanol solution. Subsequently, the top compartment of the chip was filled with a Matrigel^®^ solution of 100 µg/mL (in PBS) and incubated for 30 min at a temperature of 37 °C in the cell culture incubator. Afterwards, the chip membrane was thoroughly rinsed with A549 medium, and the A549 cells were seeded in the top channel at a density of 150,000 cells/cm^2^. The bottom channel was filled with PBS during the Matrigel^®^ solution incubation step, then filled with A549 medium and kept closed throughout cell seeding and cultivation. After a 2 h period of static culture in the incubator, the chips were either connected to the microfluidic setup or a simulated microgravity device. For the experiments, the medium was equilibrated for 24 h in a cell culture incubator before use to reduce bubble formation during chip incubation.

### 2.6. Cell Cultivation Using Chip Devices

**Figure 2:**
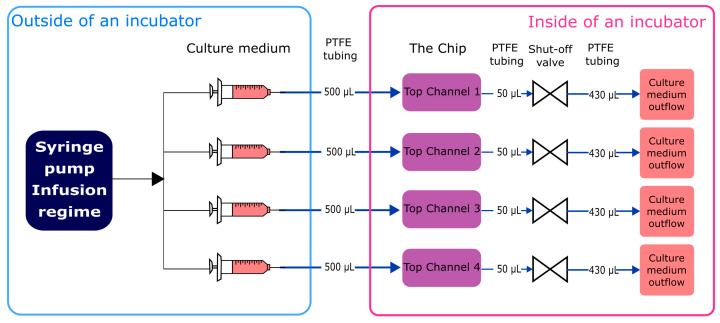
Schematic representation of custom-made jig.

Non-microgravity control tests were run using a custom-made jig (Figure 2). Culture medium was infused in the system at a flow rate 1 µL/min (generating 0.0004 dyn/cm^2^ shear stress) using a syringe pump (ISPLab02, Baoding Shenchen Precision Pump Co. Ltd., Baoding, China) and cultivated for 7 subsequent days. Inflow and outflow connections from top channels of the chip were ensured with polytetrafluoroethylene (PTFE) tubing (800 µm inner diameter (i.d.)) (Darwin microfluidics, Paris, France) and 1.5 cm pieces of Pharmed^®^ BPT rubber tubing (800 µm i.d.) (Saint-Gobain, Courbevoie, France) for connection to mini-Luer connectors (microfluidic ChipShop, Jena, Germany). Integrated shut-off valves (Darwin microfluidics, Paris, France) were integrated in between the inlet tubing path to avoid microbubble generation in the chip channels during exchange of the culture medium. The outflow was collected in 15 mL conical tubes (Sarstedt, Nümbrecht, Germany). All components were organized and fixed in place using laser-cut poly(methyl methacrylate) (PMMA) plates.

**Figure 3:**
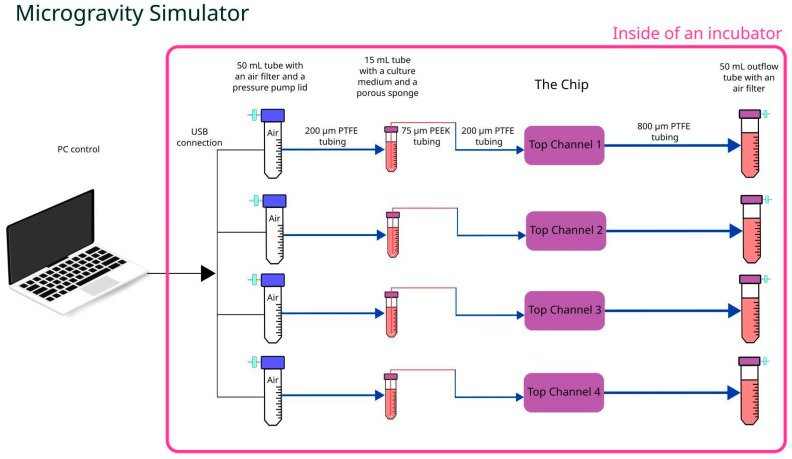
Schematic representation of microgravity setup.

Microgravity tests were run using a simulated microgravity machine combined with Cellbox Labs proprietary pumping solution to substitute syringe pumps and ensure culture medium infusion in the top channels of the chip at a flow of 1 µL/min (see Figure 3). Similarly to previously, 800 µm i.d. PTFE tubing in combination with rubber tubing and Luer male plugs were used to connect the chip’s top channels with either the pressure-driven culture medium pumping solution or with 50 mL conical tubes (Sarstedt, Germany) for outflow collection. Due to constant rotation, an additional 15 mL tube (Sarstedt, Germany) with a rigid capillary tubing retaining porous sponge at the bottom of the tube was installed in the inflow line, ensuring constant supply of the culture medium regardless of its position. Simultaneously, the same capillary tubing (20 cm long PEEKsil with a 75 µm i.d.) (IDEX Health & Science, Oak Harbor, WA, USA) reduced culture medium flow down to a rate similar to that used in normogravity experiments. The whole setup including chip, pressure pumps, and collection tubes was attached to the inner frame using holders or adhesive tape, then clinostat frames were balanced out to ensure uninterrupted and smooth rotation.

### 2.7. Brightfield Microscopy

Brightfield microscopy was performed using a Leica DM IL microscope (Leica Microsystems, Wetzlar, Germany) equipped with a 10×/N.A. 0.25 objective. A Leica ICC50 HD camera and Leica Application Suite EZ software 3.4.0 (Leica Microsystems, Germany) were used for imaging purposes.

### 2.8. Immunofluorescence Microscopy

For immunocytochemistry, membranes were fixed within the chips for 15 min using 4% paraformaldehyde and rinsed with PBS three times for five minutes. After fixation, a 20 min permeabilization step was performed using 0.2% Triton-X100 (T8787, Sigma Aldrich, St. Louis, MO, USA). After permeabilization, membranes were extracted from the device and blocked with Superblock (37580, ThermoFisher, Waltham, MA, USA) for 2 h at room temperature. After blocking, cells were incubated with primary antibodies (Anti-EpCAM, sc-25308, SantaCruz Biotechnology, Dallas, TX, USA and anti-CD133, NB120-16518, NovusBiologicals, Centennial, CO, USA) at the concentrations 1:50 and 1:100, respectively. Antibodies were diluted in the blocking buffer and incubation was performed overnight at +4 °C. The next day, membranes were washed with PBS-Tween20 (0.01%) three times for five minutes and incubated with respective secondary antibodies (Alexa Fluor 488 (Ab150113, Abcam, Cambridge, UK) and Alexa Fluor 647 (Ab150079, Abcam) diluted 1:300) for 2 h at room temperature. Subsequently, cells were washed with PBS-Tween20 (0.01%), stained with Actin-Red (R37112, ThermoFisher) and DAPI (D8417-1MG, Sigma Aldrich, St. Louis, MO, USA), then mounted with ProlongGold (P10144, Invitrogen, Waltham, MA, USA). Samples were imaged within 24 h after staining.

Confocal microscopy was performed using a Leica TCS SP8 laser scanning confocal microscope (Leica Microsystems, Wetzlar, Germany). Four laser lines were used: diode (405 nm) for DAPI, argon (488 nm) for AlexaFluor488, DPSS (561 nm) for actin red, and HeNe (633 nm) for AlexaFluor647. Z-stacks were scanned using 40×/N.A. 1.25 objective. Images and Z-stack maximum projections were then processed using Leica Application Suite X software (Leica Microsystems, Germany). Three-dimensional Z-stack reconstruction was performed using LAS X 3-D Viewer (Leica Microsystems, Germany).

### 2.9. CCK8 Viability Test

Cell viability was measured using a Cell Counting Kit-8 (96992, Sigma Aldrich, St. Louis, MA, USA).

For the plate, the cell culture medium was removed and 200 µL of 1:10 diluted CCK-8 solution was added to each well. For the chips, they were disconnected from the fluidic setup, 50 µL of 1:10 diluted CCK-8 solution was added to the top channel, and 50 µL was added to the bottom channel. Incubation with CCK-8 was performed for 1 h at 37 °C in a humidified atmosphere containing 5% CO_2_, followed by collection of CCK-8 solution from the channels or wells and absorbance detection at 450 nm by microplate reader (Victor 3V, Perkin-Elmer, Waltham, MA, USA).

### 2.10. Data Analysis

Data are expressed as the mean ± standard error (SD). Data analyses were performed by using GraphPad Prism software version 10.0 (GraphPad, San Diego, CA, USA). Data were analyzed by the Kruskal–Wallis test followed by Dunn’ s test for multiple comparison correction. Differences were considered statistically significant at a *p*-value < 0.05.

## 3. Results

During this study, validation of the microgravity simulation device was performed, showing time-averaged acceleration calculations as well as demonstrating the effects of microgravity on cell morphology and viability when cultivated in static (non-continuous flow) conditions. Evaluation of the organ-on-chip system with continuous flow was first performed under normal gravity showing characteristic cell morphology, followed by the testing of this system and assessing cell viability under microgravity compared to static cultures.

### 3.1. Validation of Microgravity in Simulated Microgravity Machine

**Figure 4:**
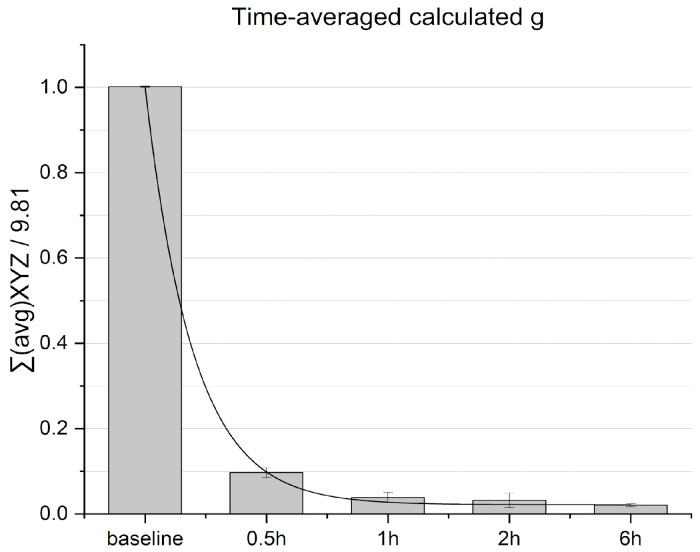
Time-averaged calculated g separated within time intervals. Normalized baseline is calculated from reads at stationary condition.

Prior to conducting the microgravity experiments, validation of the microgravity simulation system was performed by recording acceleration values and calculating the time-averaged g. Time-averaged acceleration decayed within an hour and continued to decrease to reach 0.02 g ± 0.003 values within 6 h, indicating averaged nullification of the gravity vector over time (see Figure 4).

The value obtained within 6 h is similar to that obtained in systems reported elsewhere [13], corresponding to defined microgravity requirements.

**Figure 5:**
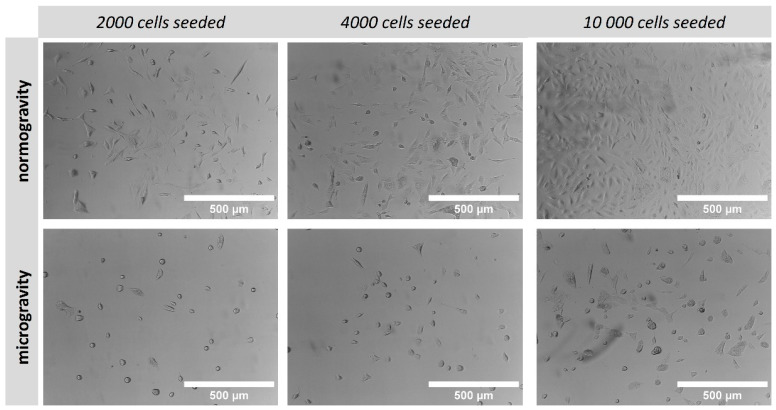
Brightfield microscopy images of A549 cells cultivated on 24-well cell culture plate under normogravity and microgravity. Scale bar: 500 μm.

In order to compare cell growth under normal gravity and microgravity conditions, A549 cells were seeded on a 24-well plate in various densities and cultivated for 7 consecutive days. Cell morphology was observed under brightfield microscopy and differences between normogravity/microgravity conditions are compared in Figure 5. An obvious reduction in cell growth was observed during microgravity regardless of the initial seeding density when comparing with cells grown under normal gravity. Simultaneously, cell morphology was also altered when cultivated under microgravity conditions, leading to more round-shaped cells (Figure 5).

### 3.2. Lung Cancer Chip Development and Exposure to Microgravity

Seeding of A549 cells and lung cancer chip model development were performed as described in the Materials and Methods section. A simple lung cancer-on-chip model from stable cell lines was developed to compare our results with previously published results of the A549 cell line’s behavior in microgravity. Due to microgravity system limitations, four dedicated pumping units could be fit within the rotating volume; therefore, in order to perform simultaneous cultivation of multiple organs, a simplified lung cancer model without vascularization was created by seeding cells in the top channels only; the bottom channels meanwhile were filled with a cell culture medium and the channels were plugged to ensure fluid flow solely across the A549 cells. The non-microgravity setup mimicked cell culturing in microgravity, meaning that cells were seeded and medium flow was applied only in the top channel. Additionally, a less complex organ model allowed us to assess the microgravity setup and scope out potential for system improvements.

**Figure 6:**
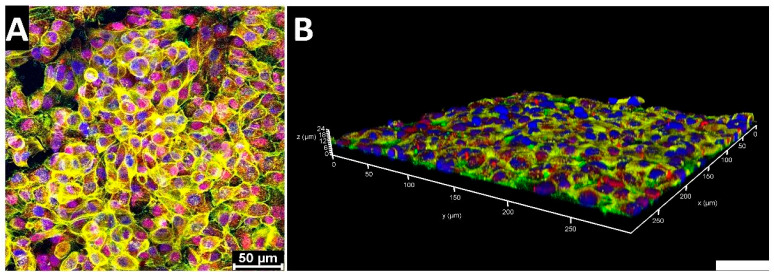
Immunofluorescence images of A549 cells on chip at normal gravity: (**A**) maximum projection of A549 cell layer (Z-stack); (**B**) 3D reconstruction of the A549 cell layer. Blue—nuclei (DAPI); yellow—F-actin; green—EpCAM; red—CD133. Scale bar: 50 μm.

Firstly, to confirm an A549 cell-specific population on top of the membrane, we performed immunofluorescence using previously described potential cancer-stem-cell-specific markers CD133 [3] and EpCAM [14] (see Figure 6). A549 cells exhibited their characteristic morphology with a high nuclei-to-cytoplasm ratio, while forming a dense, multilayered arrangement. Immunocytochemical staining identified the specific localization of the key cellular markers: EpCAM was consistently observed on the cell membranes, providing a distinctive outline of the cell periphery, while CD133 was detected within the cytoplasm as well as in the nuclei of some cells.

### 3.3. Effect of Microgravity on A549 Cell Viability

**Figure 7:**
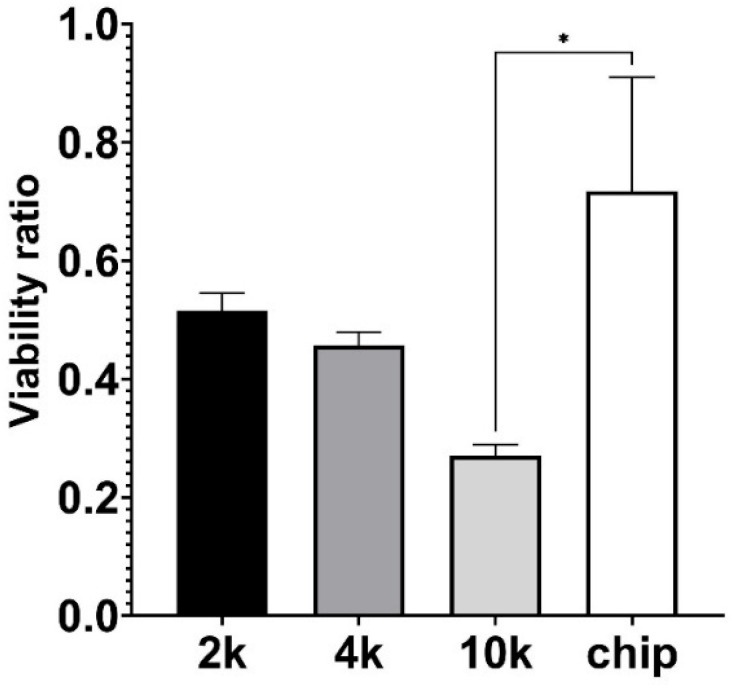
Effect of microgravity on A549 cell viability, performing CCK8 metabolic activity measurement. “2 k”, “4 k”, and “10 k” denotes cells cultivated on a 24-well plate with initial seeding density 2000, 4000, and 10,000 cells per well, respectively; “chip” corresponds to cells grown in OOC setup using continuous flow. Viability ratio is calculated by comparing CCK8 metabolic activity measurements under microgravity versus normal gravity. Significant difference is marked with asterisk (* *p* = 0.0112).

To evaluate the effect of microgravity on the cell viability, the CCK8 colorimetric assay was used as an indicator of cell metabolic activity and cell viability testing was performed after 7 consecutive cultivation days for both the cell culture plate and the chip under both normogravity and microgravity conditions (as seen in Figure 7). Data are expressed as the ratio between cell viability in microgravity against viability in normal gravity conditions. Microgravity decreased the viability and metabolic activity of cells. At the same time, the cell viability ratio was less affected in the organ-on-a-chip setup with continuous flow when compared to the static-conditions culture plate with an initial seeding density of 10,000 cells per well (*p* = 0.0112) (Figure 7).

## 4. Discussion

Research combining lung cancer-on-chip models in continuous flow with simulated microgravity devices has not been published so far; however, such studies offer a unique environment to study cell physiology, as it has been shown that microgravity exposure causes significantly larger damage to the lung epithelial barrier compared to normal gravity in experiments aboard parabolic flight campaigns [15]. In addition, the use of “3D” ground-based microgravity facilities equipped with OOC solutions offers an advantage over the use of traditional vessels and flasks, where complex fluid motions and extended culture areas could limit the availability of high-quality observations of biological processes altered exclusively by microgravity effects [16].

We observed a reduction in A549 cell viability under microgravity with continuous flow (Figure 7), which is consistent with our study and earlier studies that investigated A549 cells in static microgravity conditions [17]. The behavior of cells in static or dynamic conditions in microgravity can differ due to the lack of shear stress. Microgravity reduces the gravitational load on cells, leading to mechanical unloading and modifications to cytoskeletal structures, such as microtubules and actin filaments. However, the lack of shear stress in microgravity can also affect cell behavior, as shear stress is known to play a crucial role in regulating cell morphology, proliferation, and adhesion [2]. Therefore, the absence of shear stress in microgravity can lead to different cell behaviors compared to cells in static or dynamic conditions on Earth.

In a separate study, carcinoma cells were observed under a simulated microgravity system combined with OOC. The findings reveal that microgravity fostered the formation of multicellular spheroids, a 3D structure that mimics the in vivo tumor environment, while hypergravity suppressed their development. Furthermore, microgravity altered gene expression patterns related to cell proliferation, adhesion, and apoptosis, suggesting a profound modification of cellular pathways. The analysis of protein expression also indicated a significant modulation in response to altered gravity [18]. Nonetheless, it should be noted that in this study, the chips were maintained in a non-flow state under microgravity conditions, thus eliminating the shear stress typically encountered during cultivation. Moreover, the aforementioned condition was sustained for a mere 2 h period, in contrast to our experimental setup which entailed a 48 h continuous exposure to shear stress. OOC models in microgravity are confronted with unique challenges, including the absence of shear stress—a factor that can significantly influence cellular behavior and yield divergent outcomes when compared to cells cultured under static or dynamic conditions on Earth [19]. Consequently, the system introduced in this study has addressed a longstanding issue, thereby paving new pathways for research into cancer and various diseases under dynamic microgravity conditions.

## 5. Conclusions

In conclusion, this study demonstrates the successful development of a system capable of providing continuous flow in organ-on-a-chip (OOC) models within an RPM, thereby paving a new pathway for research under dynamic microgravity conditions. Validation of microgravity was demonstrated for the 3D MSRC system employed in this study. The results show that A549 cell viability decreased under microgravity in continuous flow in comparison to normogravity under continuous flow, although cell viability in microgravity conditions was less affected in the OOC model compared to static conditions.

## 6. Patents

The Cellbox Labs chip designs, manufacturing materials, and fabrication and bonding technology are patent-pending, and have been filed as European Patent Office (EP) and Patent Cooperation Treaty (PCT) applications EP4198119A1 and WO2023111127A1, respectively. Subsequently, accurate details of the fabrication and bonding parameters cannot be disclosed until the granting of the patent.

The custom-developed pumping technology is subject to patent application LVP2023000115 by Cellbox Labs; subsequently, disclosure of the pumping unit designs is not possible for this publication.

## Data Availability

The datasets used and analyzed during the current study are available from the corresponding author on reasonable request.
